# The Predictive Role of Contemporary Filial Piety and Academic Achievement on Multidimensional Emotional Intelligence Among Chinese Undergraduates

**DOI:** 10.3390/jintelligence13070081

**Published:** 2025-07-06

**Authors:** Longlong Zhao, Xiaohui Zhang

**Affiliations:** School of Marxism, Nanjing Institute of Technology, Jiangning District, Nanjing 211167, China; Nanjing Institute of Technology, Jiangning District, Nanjing 211167, China; j00000003479@njit.edu.cn

**Keywords:** contemporary filial piety, emotional intelligence, academic achievement, Confucian values, socioemotional development

## Abstract

This study investigates the quantitative relationship between the four dimensions of emotional intelligence and the two types of contemporary filial piety, academic achievement in a Chinese university setting. Based on a sample of 240 Chinese undergraduates, the regression analysis was employed to examine how academic achievement and the two types of contemporary filial piety, namely Pragmatic Obligation (PO) and Compassionate Reverence (CR), relate to four dimensions of emotional intelligence—Self-Emotional Monitoring (SEM), Emotional Utilization (EU), Social Competence (SC), and Others’ Emotional Appraisal (OEA). Results revealed that CR, PO, and Grade Point Average (GPA) predicted emotional intelligence positively and significantly. Notably, PO was the strongest predictor of emotional intelligence compared to CR and GPA. These findings advance theoretical understanding in two aspects. Firstly, they challenge the traditional dichotomy of filial piety by demonstrating that both CR and PO serve as cultural resources enhancing emotional competencies. Afterwards, the study bridges collectivistic values by filial piety with emotional intelligence, offering a culturally nuanced framework for interpreting academic success in Confucian societies.

## 1. Introduction

Filial piety is a Confucian value in Chinese culture that prescribes children’s duties of respect, care, and support toward their parents (Guo et al. 2022). It provides the moral foundation for parent–child relations in China, shaping socialization and individual development (Guo et al. 2022). Most researchers conceptualize filial piety as two complementary dimensions: Reciprocal filial piety (RFP), based on genuine affection and gratitude arising from warm, equal parent–child interactions, and Authoritarian filial piety (AFP), based on duty and obedience to parental authority (Guo et al. 2022; Bedford and Yeh 2019). Both dimensions coexist and have culturally unique implications: for example, research suggests that RFP and AFP can sometimes promote the same outcomes in Chinese youths, even if their motives differ (Guo et al. 2022).

Since the 21st century, alongside the gradual decline of the Authoritarian filial piety (AFP) in China, Lum et al. (2016) proposed a novel framework for reclassifying filial piety, distinguishing it into Compassionate Reverence (CR) and Pragmatic Obligation (PO). This approach demonstrates a high degree of alignment with the contemporary realities of filial piety development in China.

In a contemporary Chinese context, cultivating filial piety is thought to contribute to children’s emotional development: the affectionate care and mutual support in Reciprocal filial piety (RFP) may foster empathy and Regulation of Emotion, while the discipline and duty in AFP may encourage self-control and attunement to others’ expectations (Guo et al. 2022).

Then, emotional intelligence (EI) can be defined in two distinct ways: as an ability or as a trait (Bereded et al. 2025). The ability model of EI treats EI as a set of cognitive–emotional skills—such as accurately perceiving or managing emotions—typically measured with performance tests (Salovey and Mayer 1990). In contrast, the trait model conceives EI as a constellation of self-perceived emotional competencies and dispositions (often measured through self-report) (Bereded et al. 2025). And the trait model typically includes facets such as appraisal of one’s own emotions, appraisal of others’ emotions, Regulation of Emotion or self-control, and using emotions to facilitate thinking and action (Bereded et al. 2025).

For example, Chen et al. (2018) distinguished four EI facets—Self-Emotional Appraisal, Others’ Emotional Appraisal, Regulation of Emotion, and Use of Emotion—in their study of young adults. In this study, we adopt the trait EI perspective, using a self-report scale (a 19-item Emotional Intelligence Scale) to capture students perceived emotional abilities. This approach aligns with Petrides and Furnham’s (2001) conceptualization of trait EI as part of personality, and it differs from ability-based EI tests (e.g., Mayer–Salovey–Caruso Emotional Intelligence Test) in that there are no objectively right/wrong answers (Bereded et al. 2025).

In addition, emotional intelligence (EI) is increasingly understood as a multidimensional construct comprising distinct but interrelated abilities such as self-monitoring, emotional usage, social functioning, and emotional appraisal of others (Petrides and Furnham 2001; Chen et al. 2018). Analyzing these dimensions separately offers a more nuanced view of how specific psychological and cultural factors influence students’ emotional functioning.

Afterwards, filial piety may serve as a sociocultural context for emotional development. Contemporary filial piety’s two facets could enhance EI through different pathways. More specifically, Compassionate Reverence (CR)—rooted in genuine affection and empathy toward parents—might nurture skills like sensitivity to others’ emotions and emotional understanding, by encouraging warm family interactions (Lum et al. 2016). Pragmatic Obligation (PO)—emphasizing duty, care, and self-discipline in supporting parents—may foster emotional regulation and self-control, as children learn to manage their own needs to meet familial obligations (Lum et al. 2016). This integrative view is consistent with prior research suggesting that both Reciprocal/Affectionate and Authoritarian/Duty-based filial attitudes can positively contribute to personal development (e.g., emotional regulation and empathy) in Chinese youth. By explicitly linking these cultural values to emotional competencies, our study extends existing theories of EI into a Confucian cultural framework.

Furthermore, strong EI in students is linked to better academic and life outcomes: emotionally intelligent learners exhibit greater resilience, better stress management, and higher academic engagement (Bereded et al. 2025). Empirical work shows a positive relationship between EI and academic success, indicating that students with higher EI tend to achieve higher grades and learning outcomes (Bereded et al. 2025).

Notably, research in other cultural contexts echoes the positive connection between EI and academic success. In Western countries, numerous studies have found that students with higher emotional intelligence tend to achieve better academic outcomes (Bereded et al. 2025). For example, a recent meta-analysis confirmed that EI is a moderate-to-strong predictor of academic performance across diverse samples (Quílez-Robres et al. 2023). In the Republic of Korea and Japan—educational systems similarly influenced by Confucian values—emerging evidence also links EI to students’ scholastic adjustment and success.

In addition, Sung (2010) qualitatively showed that cultural context (East Asian vs. Western parenting practices) can shape adolescents’ emotional intelligence. Likewise, in a Japanese university sample, McEown et al. (2024) found that trait EI fosters greater academic engagement and lowers burnout, suggesting emotional competencies support learning in Asian settings.

Given the centrality of family in Chinese culture, filial piety may be an important determinant of emotional development. The affectionate parent–child bonds inherent in Reciprocal filial piety likely provide rich learning experiences for recognizing and managing emotions: children who feel loved and respected by their parents may have more opportunities to practice empathy and Regulation of Emotion (Chen et al. 2018). Indeed, Chen et al. (2018) found that Taiwanese college students’ Reciprocal filial piety was positively associated with all four EI dimensions (Self-Emotional Appraisal, Others’ Emotional Appraisal, Regulation of Emotion, and Use of Emotion). Consistent with this, other studies note that higher levels of RFP are linked to greater emotional intelligence and cognitive flexibility (Liu and Chong 2024). In contrast, Authoritarian filial piety (AFP) emphasizes obedience and suppression of personal desires (Guo et al. 2022). Although less frequently studied, AFP may still relate to aspects of EI. For example, the self-control and attentiveness to parental cues required by AFP could enhance children’s emotional self-regulation and sensitivity to others’ feelings.

Prior research in Chinese samples suggests that Reciprocal filial piety (RFP) and AFP can sometimes both contribute positively to personal outcomes (Guo et al. 2022). Together, these observations imply a rationale for testing whether RFP and AFP are positively associated with EI dimensions in Chinese students. However, most existing filial piety and EI studies have focused on Taiwanese or non-mainland samples (e.g., Chen et al. 2018) or have considered RFP alone. We are unaware of any quantitative research examining how the two types of contemporary filial piety (Compassionate Reverence and Pragmatic Obligation, advocated by Lum et al. (2016)) relate to multiple EI facets among undergraduates in Mainland China. Addressing this gap, the present study examines associations between the two types of contemporary filial piety advocated by Lum et al. (2016) and four dimensions of emotional intelligence in a sample of Mainland Chinese college students.

Furthermore, academic performance, often measured by Grade Point Average (GPA), is another important outcome linked to family values and emotional skills. In Chinese education, academic achievement is highly valued and even seen as a way of honouring one’s parents (Guo et al. 2022). Families instill in students the idea that doing well in school repays parental sacrifices. Given the strong connection between schooling and family expectations in China, students’ academic success may be related to their emotional competencies. Indeed, a robust literature has found that EI and academic performance are positively correlated (Bereded et al. 2025). For example, emotionally intelligent students have better motivation and learning strategies, contributing to higher grades (Bereded et al. 2025). However, these studies typically view EI as a predictor of academic success.

In contrast, little research has tested whether a higher-Grade Point Average (GPA) might predict greater emotional intelligence. This reverse perspective is novel. On the one hand, students with high academic achievement may have developed disciplined study habits and stress-management skills that overlap with EI competencies (such as self-control and emotional use). On the other hand, excelling academically might build confidence and social opportunities that facilitate emotional awareness. Whatever the mechanism, exploring Grade Point Average (GPA) as a predictor of EI has not been conducted in Chinese undergraduates. Thus, the present study also investigates whether GPA is positively connected with each EI dimension.

In summary, filial piety is a culturally unique construct in China that likely influences individuals’ emotional development through family interactions. Meanwhile, Grade Point Average (GPA) is a crucial indicator of success in Chinese society and may be intertwined with emotional skills. The current research aims to fill this gap because the literature lacks quantitative tests of these relationships in Mainland Chinese students. Specifically, we examine how Compassionate Reverence (CR) and Pragmatic Obligation (PO) relate to the four facets of emotional intelligence (Self-Emotional Appraisal, Others’ Emotional Appraisal, Regulation of Emotion, and Use of Emotion) and whether GPA relates to these EI facets. According to emotional socialization theory, family practices and values (such as filial piety) provide a context for learning emotional skills. Thus, both the affectionate bonds of CR and the disciplined responsibilities of PO could foster different facets of EI by encouraging empathy, self-regulation, and sensitivity to others’ feelings. Based on theory and prior findings, we propose the following focused hypotheses:

**H1.**  *Both Compassionate Reverence (CR) and Pragmatic Obligation (PO) will predict each dimension of students’ emotional intelligence positively and significantly.*

**H2.**  *Grade Point Average (GPA) will predict each dimension of students’ emotional intelligence positively and significantly.*

**H3.**  *Among the predictors, Pragmatic Obligation (PO) will show the strongest contribution to predicting students’ emotional intelligence.*

## 2. Materials and Methods

### 2.1. Participants

A total of 312 questionnaires were distributed in this study, and 286 (91.67%) were recovered, of which 240 (83.92%) were valid questionnaires. The participants of this study were mainly college students from two universities in Nanjing (China). In terms of the number of valid questionnaires, there were 84 male participants (35%) and 156 female participants (65%).

Secondly, participants were mostly between 18 and 23 years old (28% in the 21~23 range). The sample’s age distribution was 18 to over 33, with the majority in their early 20s.

Then, 36.2% of participants whose Grade Point Average (GPA) is 3.51~4.00, 32.9% of participants’ Grade Point Average is 4.01~4.50, and 21.2% of participants’ Grade Point Average is 3.00~3.50 (the full mark of the GPA is 5.00). Furthermore, the Mean and Standard Deviation of participants’ Grade Point Averages are “3.19” and “0.94” (“1”: below 3.00, “2”: 3.00~3.50, “3”: 3.51~4.00, “4”: 4.01~4.50, “5”: 4.51 and above), respectively.

Although GPA was collected in categories (by range) for descriptive purposes, we treated GPA as an approximately continuous variable in analyses. Each student’s GPA was converted to a numeric value on a five-point scale (with higher scores reflecting better academic performance). Treating GPA as continuous is justified given that it represents an underlying continuous achievement level (0–5 scale). This approach preserves information and is consistent with prior research using GPA in correlation/regression analyses.

### 2.2. Instruments

Three questionnaires were employed in this study, and the first questionnaire was mainly used to collect the participants’ basic information, such as gender, age, and the Grade Point Average (GPA). As the college students who filled out the questionnaire were all Chinese, the first questionnaire did not collect the nationality of the participants.

Then, the second questionnaire is a 19-item Emotional Intelligence Scale (EIS-19) revised by Huang et al. (2008) according to the 33-item Emotional Intelligence Scale (EIS-33) created by Schutte et al. (1998). The EIS-19 consists of 19 questions, all of which are positively scored, and each of which is scored on a five-point Likert scale, where “1” stands for “completely inconsistent”, “5” stands for “completely consistent”.

In addition, the EIS-19 is a self-report trait EI scale adapted from Schutte et al. (1998), covering Self-Emotional Monitoring (SEM), Others’ Emotional Appraisal (OEA), Social Competence (SC), and Emotional Utilization (EU). Moreover, the Self-Emotional Monitoring (SEM), Social Competence (SC), and Emotional Utilization (EU) contain five questions each, while the Others’ Emotional Appraisal (OEA) contains only four questions.

More specifically, the 1st, 26th, 19th, 12th, and 21st questions belong to Self-Emotional Monitoring (SEM); the 25th, 18th, 32nd, and 29th questions belong to Others’ Emotional Appraisal (OEA); the 30th, 16th, 14th, 4th, and 3rd questions belong to Social Competence (SC); and the 6th, 9th, 20th, 8th, and 7th questions belong to Emotional Utilization (EU). Meanwhile, the 5th, 28th, and 33rd questions were deleted by Huang et al. (2008). And the serial numbers of the questions mentioned above correspond to the serial numbers of the questions in the EIS-33.

Finally, the third questionnaire was the 10-item contemporary filial piety scale (CFPS-10) designed by Lum et al. (2016). The scale consists of a total of 10 questions, each of which is scored on a Likert five-point scale, where “1” stands for “Strongly Disagree” and “5” for “Strongly Agree”. The contemporary filial piety scale (CFPS-10; Lum et al. 2016) captures cultural values, dividing filial piety into Pragmatic Obligation (PO) and Compassionate Reverence (CR).

Notably, CR and PO are like the affectionate (Reciprocal) and dutiful (Authoritarian) aspects of filial piety, respectively (Lum et al. 2016). We emphasize that while traditional filial piety models (e.g., Yeh and Bedford’s dual filial piety) use terms like Reciprocal versus Authoritarian filial piety, the CFPS-10’s CR and PO are conceptually similar constructs (Lum et al. 2016).

As for PO, it contains six questions related to caregiving practices, while CR contains four questions related to emotional caregiving. More specifically, the 1st, 2nd, 3rd, 4th, 5th, and 6th questions belong to PO, while the 7th, 8th, 9th, and 10th questions belong to CR.

### 2.3. Procedure

Before data collection began, the study protocol and all materials were reviewed and approved by the Institutional Ethics Committee of Nanjing Institute of Technology. Participants were recruited voluntarily from two universities in Nanjing, Jiangsu Province, using a convenience sampling method. All participants provided informed consent after being briefed on the study’s purpose, confidentiality assurances, and their right to withdraw at any time.

Then, participants self-reported their Grade Point Average (GPA), which was initially collected in categorical intervals for descriptive purposes but later converted to a continuous five-point scale for analysis, consistent with established practices in prior research. The two focal constructs—contemporary filial piety and emotional intelligence—were measured using validated Chinese versions of the CFPS-10 and EIS-19 instruments.

We employed a convenience sampling strategy (recruiting volunteers from two universities) due to practical constraints. Although convenience sampling may limit generalizability, the chosen universities were typical of the region, and our sample of 240 undergraduates still provides valuable insights as an exploratory study in this context. Additionally, this sampling method has a limitation, but it is common in initial studies of this kind.

These questionnaires were issued at the end of March 2025 and were recycled in early April 2025. After the participants complete and submit the questionnaire, they can receive a souvenir worth 1 yuan. After about two weeks, we distributed 312 questionnaires and collected 286 questionnaires (91.67%).

Subsequently, we sorted out the collected data. After removing the invalid questionnaires, we found that 240 (83.92%) of the questionnaires collected this time were valid. Afterwards, assisted by Stata SE16.0 (a software for data statistics and analysis), we conducted a detailed study on the quantitative relationship between the types of current filial piety or the Grade Point Average and the four dimensions of emotional intelligence with the help of partial correlation analysis and regression analysis. Finally, based on the results of the data analysis, this article was written.

### 2.4. Data Analysis

Stata SE16.0 (a software for data statistics and analysis) for MacBook was employed to implement the data analysis task. Firstly, we tested the Cronbach coefficient (α) of the questionnaire. The Cronbach coefficients (α) of CFPS-10 and EIS-19 are 0.896 and 0.894, respectively, and the Cronbach coefficients (α) of Self-Emotional Monitoring (SEM), Emotional Utilization (EU), Social Competence (SC), and Others’ Emotional Appraisal (OEA) are 0.826, 0.816, 0.808, and 0.779, individually. As presented in the results, both CFPS-10 and EIS-19 have an acceptable performance in terms of consistency.

Then, we conducted partial correlation analyses to examine the bivariate relationships between each contemporary filial piety dimension (PO, CR) and each EI dimension (Self-Emotional Monitoring, Others’ Emotional Appraisal, Social Competence, and Emotional Utilization) while controlling for the influence of the other predictors.

Specifically, we controlled for GPA and the other contemporary filial piety variable in each partial correlation. This allowed us to isolate the unique association of Compassionate Reverence (CR) with an EI subscale independent of Pragmatic Obligation (PO) and GPA. We chose partial correlations instead of simple Pearson correlations because Pragmatic Obligation (PO), Compassionate Reverence (CR), and GPA are interrelated, and we wanted to account for those interrelations when testing our hypotheses.

The correlation results were explained in three aspects, namely a strong correlation (the coefficient ≥ 0.5), a medium correlation (the coefficient ≥ 0.3 and the coefficient < 0.5), and a weak correlation (the coefficient < 0.3).

Next, based on the results of partial correlation analysis, we built five regression models. In the five regression models, GPA and the two types of contemporary filial piety (PO and CR) were taken as independent variables, and the four dimensions of emotional intelligence were regarded as dependent variables, respectively.

As presented in the results, the quantitative relationship between the four dimensions of emotional intelligence (Self-Emotional Monitoring, Others’ Emotional Appraisal, Social Competence, and Emotional Utilization) and GPA or the contemporary filial piety (PO and CR) types was further explored.

## 3. Results

### 3.1. Partial Correlation Analysis

We conducted partial correlation analyses to examine the unique association of each predictor (CR, PO, GPA) with each dimension of emotional intelligence, controlling for the other two predictors in each analysis. The results indicated that both CR and PO were significantly positively correlated with all four EI dimensions, with PO showing stronger associations overall. For instance, PO exhibited strong correlations with Emotional Utilization (r = 0.60, *p* < 0.01) and Social Competence (r = 0.59, *p* < 0.01), while CR was moderately correlated with these outcomes (see Table 1).

Similarly, GPA was moderately correlated with Self-Emotional Monitoring (r = 0.32, *p* < 0.01) and Emotional Utilization (r = 0.36, *p* < 0.01), and weakly correlated with Social Competence and Others’ Emotional Appraisal (see Table 2). These findings suggest that academic achievement may play a supplementary role in shaping emotional intelligence, relative to the stronger influence of filial piety variables.

In addition, Table A1 presents the statistical description of the main variables (see Appendix A).

### 3.2. Regression Analysis

Assisted with regression analysis, the quantitative relationship between the two types of contemporary filial piety and the four dimensions of emotional intelligence was explored. In the meantime, five regression models were built. In this paper’s first four regression models, the two types of contemporary filial piety, namely Pragmatic Obligation (PO) and Compassionate Reverence (CR), are regarded as independent variables. Meanwhile, the four dimensions of emotional intelligence, Self-Emotional Monitoring (SEM), Emotional Utilization (EU), Social Competence (SC), and Others’ Emotional Appraisal (OEA), were treated as the dependent variable. Then, in the fifth regression model, emotional intelligence was treated as the dependent variable, while GPA, PO, and CR were regarded as the independent variables. Prior to regression, we assessed multicollinearity among the independent variables (PO, CR, and GPA). All variance inflation factor (VIF) values were well below 5 and tolerance values above 0.5, indicating no multicollinearity problems. This suggests the two contemporary filial piety variables, although correlated, each contribute unique variance to the models.

As presented in Table 3, there is a significant positive association between Self-Emotional Monitoring (SEM) and Pragmatic Obligation (PO). In Model 1, compared with Compassionate Reverence (CR), Pragmatic Obligation (PO) has a stronger association with Self-Emotional Monitoring (SEM).

Next, Table 3 presents a significant positive association between Emotional Utilization (EU) and Pragmatic Obligation (PO) or Compassionate Reverence (CR). In Model 2, compared with CR, the association between PO and Emotional Utilization (EU) is stronger.

Thirdly, as shown in Table 3, there is a significant positive association between Social Competence (SC) and Pragmatic Obligation (PO) or Compassionate Reverence (CR). In Model 3, compared with CR, the association between PO and Social Competence (SC) is stronger.

Afterwards, as shown in Table 3, there is a significant positive association between Others’ Emotional Appraisal (OEA) and Pragmatic Obligation (PO) or CR. In Model 4, the association between Compassionate Reverence (CR) and Others’ Emotional Appraisal (OEA) is stronger compared with PO. 

As shown in Table 3, a significant positive association exists between emotional intelligence and Pragmatic Obligation (PO) or Compassionate Reverence (CR). In Model 5, the association between PO and emotional intelligence is stronger compared with CR. In a nutshell, based on the first four results presented above, both CR and PO could positively predict each dimension of emotional intelligence.

Assisted by the regression analysis, the quantitative relationship between GPA and the four dimensions of emotional intelligence was explored. Then, five regression models were built. In the first four regression models established, GPA was regarded as an independent variable, and the four dimensions of emotional intelligence, namely Self-Emotional Monitoring (SEM), Emotional Utilization (EU), Social Competence (SC), and Others’ Emotional Appraisal (OEA), were regarded as dependent variables. Moreover, emotional intelligence was regarded as the dependent variable in the fifth regression model, and GPA, Pragmatic Obligation (PO), and Compassionate Reverence (CR) were considered independent variables.

As shown in Table 3, the following associations can be figured out. Firstly, there is a significant positive association between SEM and GPA. Secondly, there is a significant positive association between the EU and GPA. Thirdly, there is a non-significant association between SC and GPA. Then, there is a non-significant association between OEA and GPA. Finally, a significant positive association is found between emotional intelligence and Pragmatic Obligation (PO), Compassionate Reverence (CR), or GPA. Therefore, GPA could positively predict two dimensions of emotional intelligence.

In Model 5, compared with GPA and CR, PO has the strongest association with emotional intelligence. Additionally, among the three predictive factors, CR, PO, and GPA, GPA has the weakest association with emotional intelligence in a Chinese university setting. In short, based on the four results presented above, two parts of H2 could pass the test and be accepted.

## 4. Discussion

### 4.1. Primary Findings and the Connection with Previous Research

This study explored the role of contemporary filial piety and academic achievement (namely, Grade Point Average) in predicting emotional intelligence (EI) among Chinese undergraduates. Our findings suggest that Compassionate Reverence (CR), one of the two key dimensions of filial piety, could predict EI positively and significantly, which aligns with cultural models of socialization. This finding is consistent with socioemotional selectivity theory (Carstensen 1992), which argues that as individuals prioritize emotionally meaningful relationships, they may also develop better emotional regulation skills.

In the context of filial piety, the emotionally warm and Reciprocal nature of CR enhances empathy and emotional awareness, which are key components of EI. This finding supports previous research on emotional socialization in Confucian cultures, which emphasizes family bonds as a central mechanism for emotional development (Bedford and Yeh 2019; Chen et al. 2018).

Interestingly, Pragmatic Obligation (PO), which reflects duty-bound behaviors without emotional warmth, was a stronger predictor of EI in this sample. This result may reflect a cultural connection between the emotionally supportive aspect of filial piety (namely, CR) and more duty-driven obligations that foster emotional expression. 

It is intriguing that Pragmatic Obligation (duty-based filial piety) was an even stronger predictor of EI than Compassionate Reverence (affection-based filial piety). One possible explanation is that in Chinese culture, fulfilling pragmatic caregiving duties still involves substantial social interaction and self-discipline, which over time cultivate emotional regulation and empathy. In practice, a student who consistently cares for family members (PO) may develop patience, perspective-taking, and emotional management skills—even if the motive is duty rather than warmth. Thus, although PO lacks explicit emotional affection, it may nevertheless foster emotional competencies through the responsibilities and interpersonal experiences it entails. So, according to the above findings, H1 could be supported partly.

Our findings are different from Sánchez-Álvarez et al. (2020), who noted that emotional intelligence is often more strongly influenced by emotion-related socialization (e.g., warm familial relationships) than by purely obligatory caregiving.

Then, the role of academic achievement (GPA) in predicting EI was more nuanced. While GPA correlated with Self-Emotional Monitoring and Emotional Utilization, these associations were relatively weak, suggesting that while academic success might foster some emotional competencies, it is not a primary predictor of EI. This supports findings from MacCann et al. (2020) and Perera and Michelle (2013), who found that the relationship between EI and academic performance is generally moderate, with many variables (including emotional and social skills) influencing academic success.

However, this result contrasts with those reported by Wu and Wang (2017) and Quílez-Robres et al. (2023). One possible explanation for this discrepancy is the variation in academic performance indicators: while this study employed GPA as the primary factor, others used different elements.

Furthermore, while previous research has frequently identified emotional intelligence as a predictor of academic achievement (Ye et al. 2024), the present study offers a complementary perspective, suggesting that academic performance, as reflected by GPA, may also predict emotional intelligence. Thus, H2 was partially supported (only SEM and EU showed the predicted relationship with GPA, whereas SC and OEA did not).

Next, in Model 5, among the three predictors, Pragmatic Obligation (PO) shows the strongest contribution to predicting students’ emotional intelligence. Therefore, H3 could be supported.

These results can be interpreted through a cultural–developmental lens: filial piety practices may function as socialization mechanisms that cultivate emotional intelligence. This aligns with attachment theory and social learning theory, which suggest that supportive family interactions (as in CR) and structured responsibilities (as in PO) each contribute to children’s emotional regulation and understanding. By framing our findings in such theoretical terms, we see that filial piety may serve as a culturally specific pathway to developing EI competencies.

Our study is consistent with cross-national research showing a modest positive relationship between trait EI and academic performance (Sánchez-Álvarez et al. 2020). However, our findings also underscore the cultural specificity of these relationships.

In Chinese culture, where filial piety is a central value, familial emotional support (CR) may play a more significant role in shaping students’ emotional competencies than academic achievement alone. These findings provide a culturally grounded perspective on EI development, highlighting the importance of filial piety as a predictor of EI in Confucian societies.

These findings suggest that family socialization processes rooted in Confucian filial norms contribute to students’ emotional development in distinct ways. The stronger predictive role of PO over CR may reflect the behavioral nature of duty-based caregiving, which often involves sustained interpersonal coordination, self-regulation, and attention to others’ emotional needs. While CR is conceptually tied to emotional warmth, the structured behavioral obligations in PO may offer more consistent, practiced opportunities for cultivating emotional control and awareness. Taken together, the results support a dual-pathway framework: one emotional (CR) and one behavioral (PO), both contributing to trait emotional intelligence. GPA also demonstrated predictive value, albeit weaker, supporting the idea that academic achievement and emotional competencies may develop synergistically but not uniformly.

Overall, our findings suggest two pathways through which family context can enhance emotional intelligence: (1) an affection pathway, via warm supportive relationships (CR), and (2) a responsibility pathway, via dutiful caregiving roles (PO). In addition, academic achievement offers a third, comparatively weaker, pathway—possibly reflecting that scholastic success imparts some self-regulatory skills relevant to EI, but to a lesser extent than family socialization. Integrating these factors, we propose that the Confucian familial environment simultaneously nurtures emotional skills through love and through duty, whereas academic accomplishment plays a smaller, supplementary role.

### 4.2. Limitations

This study has four primary limitations. First, all participants were drawn exclusively from mainland China. The absence of samples from countries with distinct cultural contexts (e.g., the United Kingdom and the United States) and from culturally similar regions (e.g., the Republic of Korea and Japan) limits the generalizability of the findings across different cultural settings. As a result, the applicability of the conclusions in an international context remains constrained.

Second, while emotional intelligence is influenced by various psychological factors, including personality traits (Alghamdi et al. 2017; Leonard and Harvey 2007), this study did not collect data on participants’ personality characteristics. This omission may represent a limitation in fully capturing the complexity of the factors that shape emotional intelligence.

Third, the use of convenience sampling from a single cultural region limits the generalizability of the findings to broader or international populations. Fourth, emotional intelligence was assessed solely through self-report, which may introduce bias.

Thus, it is worth considering how these dynamics play out in other cultural contexts. In more individualistic Western societies, parent–child relationships emphasize autonomy over filial obligations, which might weaken the link between ‘duty’-based family practices and emotional skills. Conversely, other collectivist cultures (e.g., Korea, Japan) that share Confucian heritage may show patterns like China’s—indeed, some research suggests familial respect and academic diligence are tied to socioemotional adjustment in those contexts as well. Future studies should directly compare whether Compassionate Reverence (CR) and Pragmatic Obligation (PO) relate to emotional intelligence in non-Chinese samples. By engaging with these cross-cultural perspectives, we underscore that our findings are culturally situated and encourage caution in generalizing them universally.

Moreover, future research should address the limitations noted here by incorporating multi-method measures of EI (e.g., observer or performance-based assessments), expanding to rural or international samples, and assessing additional psychological constructs such as Big Five personality traits. Longitudinal or experimental designs may also help clarify the causal directionality between familial values, academic performance, and emotional competencies.

### 4.3. Practical Implications

This study tests the association between the four dimensions of emotional intelligence and the two types of contemporary filial piety or the Grade Point Average in a Chinese university setting. According to the research results, this study mainly has three practical implications.

Firstly, based on the significant positive association between emotional intelligence and the two types of contemporary filial piety (CR and PO), it might be an efficient way for parents dedicated to improving their children’s emotional intelligence by guiding them to deliver their respect and love to family members. For example, parents could hold family gratitude activities and encourage children to express their gratitude and respect to their grandparents through letters or orally.

Secondly, it may promote students’ understanding of Self-Emotional Monitoring (SEM), Emotional Utilization (EU), Social Competence (SC), and Others’ Emotional Appraisal (OEA) by offering students opportunities to play the roles of parents in family ethics dramas to improve their empathy. Thirdly, emotional intelligence might be improved by tackling the problems of filial piety cases in traditional Chinese culture. While analyzing the possible practical predicaments in filial piety cases, students’ ability to utilize, monitor, evaluate, or manage emotions could be strengthened to a large extent. Meanwhile, the excellent traditions in traditional Chinese culture could also be passed on from generation to generation.

## 5. Conclusions

This study contributes to our understanding of emotional intelligence (EI) by integrating cultural concepts of contemporary filial piety and academic achievement. We found that Pragmatic Obligation (PO) and Compassionate Reverence (CR), two dimensions of contemporary filial piety, significantly predict higher emotional intelligence, suggesting that emotionally supportive familial interactions foster EI development in Chinese undergraduates. Thus, both CR and PO could predict each dimension of students’ emotional intelligence positively and significantly (CR failed to predict SEM significantly).

Then, GPA could predict Self-Emotional Monitoring (SEM) and Emotional Utilization (EU) positively and significantly. At the same time, GPA failed to predict Social Competence (SC) and Others’ Emotional Appraisal (OEA) positively and significantly. Furthermore, the weak association between academic achievement (GPA) and EI challenges the notion that high academic performance leads to better emotional skills, as emphasized in some educational psychology models (MacCann et al. 2020).

Our findings suggest that fostering PO and CR in Chinese families may play an important role in the emotional development of youth, with implications for educational practice. Given the important role of family in shaping emotional skills, educators and policymakers might consider integrating family-based interventions that emphasize emotionally supportive familial practices, enhancing not just academic outcomes but also students’ emotional well-being.

Theoretically, this study is the first to empirically model the relationship between emotional intelligence and contemporary filial piety in a Chinese university context using regression analysis. It also reconfirms the positive link between emotional intelligence and GPA. Moreover, PO, CR, and GPA collectively explain 46.16% of the variance in emotional intelligence among Chinese undergraduates. These findings underscore the dynamic interplay between ethical emotions and psychological capacities within collectivist cultural frameworks.

What’s more, these findings suggest a synergistic improvement mechanism between emotional intelligence and contemporary filial piety in a Chinese university setting. For example, strengthening the CR and PO in family education may foster emotional intelligence. Meanwhile, improving a sense called PO in a family setting may help enhance individual empathy. Nonetheless, the international adaptability of the results presented by this research is limited, as its primary research object is the Chinese undergraduates. In the future, cross-cultural and longitudinal studies should be conducted.

This study also acknowledges several limitations. The sample was drawn from urban Chinese university students, which limits generalizability to rural or international populations. Future research should explore these relationships in diverse cultural contexts to further understand the cultural variations in the EI-academic achievement link. Additionally, a longitudinal design would help establish causal pathways and better understand how contemporary filial piety and academic success interact over time to influence emotional intelligence.

## Figures and Tables

**Table 1:** Results of the partial correlations (four dimensions of EI and two types of contemporary filial piety).

	1	2	3	4	5	6
SEM	1.000					
EU	0.528 ***	1.000				
SC	0.424 ***	0.585 ***	1.000			
OEA	0.332 ***	0.375 ***	0.425 ***	1.000		
CR	0.338 ***	0.450 ***	0.487 ***	0.415 ***	1.000	
PO	0.410 ***	0.602 ***	0.591 ***	0.380 ***	0.638 ***	1.000

Note: *** *p* < 0.01, Self-Emotional Monitoring (SEM), Others’ Emotional Appraisal (OEA), Social Competence (SC), Emotional Utilization (EU), Pragmatic Obligation (PO), and Compassionate Reverence (CR).

**Table 2:** Results of the partial correlations (four dimensions of EI and GPA).

	1	2	3	4	5
SEM	1.000				
EU	0.528 ***	1.000			
SC	0.424 ***	0.585 ***	1.000		
OEA	0.332 ***	0.375 ***	0.425 ***	1.000	
GPA	0.324 ***	0.356 ***	0.254 ***	0.190 ***	1.000

Note: *** *p* < 0.01, Self-Emotional Monitoring (SEM), Others’ Emotional Appraisal (OEA), Social Competence (SC), Emotional Utilization (EU), Pragmatic Obligation (PO), and Compassionate Reverence (CR).

**Table 3:** Results of the regression analysis.

Variable	Model 1 (β/SE)	Model 2 (β/SE)	Model 3 (β/SE)	Model 4 (β/SE)	Model 5 (β/SE)
Compassionate Reverence	0.116 (0.080)	0.181 (0.065) ***	0.185 (0.066) ***	0.287 (0.075) ***	0.187 (0.048) ***
Pragmatic Obligation	0.276 (0.082) ***	0.425 (0.066) ***	0.460 (0.068) ***	0.185 (0.077) **	0.344 (0.049) ***
Grade Point Average	0.141 (0.053) ***	0.080 (0.043) *	−0.011 (0.044)	0.007 (0.050)	0.057 (0.032) *
Adjust R-square	19.15%	38.58%	36.17%	18.42%	46.16%
Number of observations	240	240	240	240	240

Note: SE = Standard Error, *** *p* < 0.01, ** *p* < 0.05, * *p* < 0.1.

**Figure A1:**
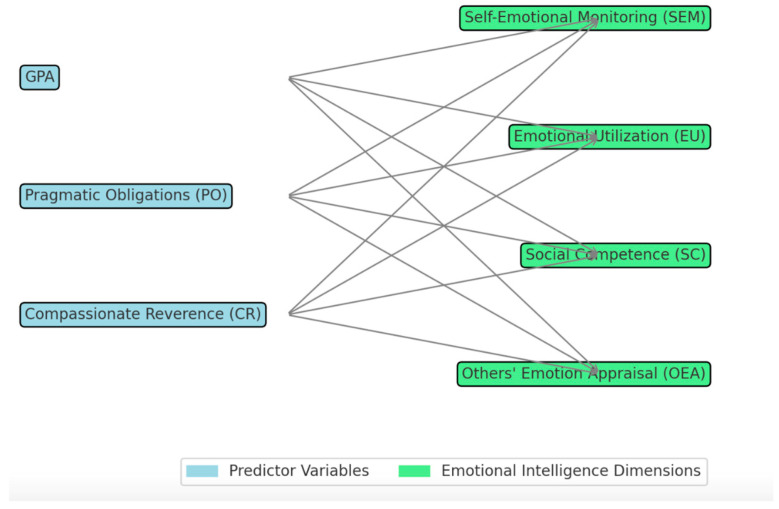
Conceptual Path Model: Predictors of Emotional Intelligence Dimensions.

## Data Availability

The raw data supporting the conclusions of this article will be made available by the authors on request.

## Appendix A

**Table A1:** The statistical description of the main variables.

Variable	Mean	Standard Deviation
Age	3.529	1.610
Self-Emotional Monitoring (SEM)	3.483	0.774
Emotional Utilization (EU)	3.734	0.720
Social Competence (SC)	3.818	0.719
Others’ Emotional Appraisal (OEA)	3.678	0.723
Compassionate Reverence (CR)	3.952	0.731
Pragmatic Obligation (PO)	4.100	0.747
Emotional Intelligence (EI)	3.679	0.564
Grade Point Average (GPA)	3.192	0.944

Note: GPA on a five-point scale from 0 (low) to 5 (high).
